# Data-driven studies of magnetic two-dimensional materials

**DOI:** 10.1038/s41598-020-72811-z

**Published:** 2020-09-25

**Authors:** Trevor David Rhone, Wei Chen, Shaan Desai, Steven B. Torrisi, Daniel T. Larson, Amir Yacoby, Efthimios Kaxiras

**Affiliations:** 1 Department of Physics, Harvard University, Cambridge, MA 02138 USA; 2 School of Engineering and Applied Sciences, Harvard University, Cambridge, MA 02138 USA

**Keywords:** Magnetic properties and materials, Two-dimensional materials, Computational science, Electronic properties and materials, Ferromagnetism

## Abstract

We use a data-driven approach to study the magnetic and thermodynamic properties of van der Waals (vdW) layered materials. We investigate monolayers of the form $\hbox {A}_2\hbox {B}_2\hbox {X}_6$, based on the known material $\hbox {Cr}_2\hbox {Ge}_2\hbox {Te}_6$, using density functional theory (DFT) calculations and machine learning methods to determine their magnetic properties, such as magnetic order and magnetic moment. We also examine formation energies and use them as a proxy for chemical stability. We show that machine learning tools, combined with DFT calculations, can provide a computationally efficient means to predict properties of such two-dimensional (2D) magnetic materials. Our data analytics approach provides insights into the microscopic origins of magnetic ordering in these systems. For instance, we find that the X site strongly affects the magnetic coupling between neighboring A sites, which drives the magnetic ordering. Our approach opens new ways for rapid discovery of chemically stable vdW materials that exhibit magnetic behavior.

## Introduction

The discovery of graphene ushered in a new era of studies of materials properties in the two-dimensional (2D) limit^1^. For many years after this discovery only a handful of van der Waals (vdW) materials were extensively studied. Recently, over a thousand new 2D crystals have been proposed^2–5^. The explosion in the number of known 2D materials increases demands for probing them for exciting new physics and potential applications^6,7^. Several 2D materials have already been shown to exhibit a range of exotic properties including superconductivity, topological insulating behavior and half-metallicity^8–11^. Consequently, there is a need to develop tools to quickly screen a large number of 2D materials for targeted properties. Traditional approaches, based on sequential quantum mechanical calculations or experiments are usually slow and costly. Furthermore, a generic approach to design a crystal structure with any desired property, although an active area of research^12–15^ and of practical significance, does not exist yet. Research towards building structure-property relationships of crystals is in its infancy^16–19^.

Long-range ferromagnetism and anti-ferromagnetism in 2D crystals has recently been discovered^20–24^, sparking a push to understand the properties of these 2D magnetic materials and to discover new ones with improved behavior^5,19,25–32^. 2D crystals provide a unique platform for exploring the microscopic origins of magnetic ordering in reduced dimensions. Long-range magnetic order is strongly suppressed in 2D according to the Mermin-Wagner theorem^33^, but magnetocrystalline anisotropy can stabilize magnetic ordering^34^. This magnetic anisotropy is driven by spin-orbit coupling which depends on the relative positions of atoms and their identities. As a result, the magnetic order should be strongly affected by changes in the structural arrangements of atoms and chemical composition of the crystal.

Chemical instability presents a crucial limitation to the fabrication and use of 2D magnetic materials. For instance, black phosphorous degrades upon exposure to air and thus needs to be handled and stored in vacuum or under inert atmosphere^35^. Structural stability is a necessary ingredient for industrial scale application of magnetic vdW materials, such as $\hbox {CrI}_3$ and $\hbox {Cr}_2\hbox {Ge}_2\hbox {Te}_6$^23,24^. In addition to designing 2D materials for desirable magnetic properties, it is important to screen for materials that are chemically stable. In our approach, we employ the calculated formation energy as a proxy for the chemical stability^36^. A recent data-driven study found that formation energy was one of the most important predictors of 2D MXene stability^37^, lining up with heuristics identified in the 2D Materials community^5,38^. To calculate the formation energy, we obtain the total energies of systems at zero temperature, and obtain the difference in total energy between the crystal and its constituent elements in their respective crystal phases. This quantity determines whether the structure is thermodynamically stable or would decompose. This formulation ignores the effects of zero-point vibrational energy and entropy on the stability.

While the formation energy provides evidence for thermodynamic stability, dynamic stability can also be assessed. By computing the phonon spectrum of the 0K structure, the presence of negative phonon frequencies demonstrates dynamic instability. All-positive frequencies demonstrate a structure stable against small perturbations, suggesting that a freestanding monolayer may be stable and experimentally accessible^5^.

The magnetic properties at finite temperatures are also important. There is growing interest in identifying 2D materials with magnetic order above room temperature for fundamental research and device applications^29,39^. Consequently, it is desirable to build a tool to screen for 2D materials which are ferromagnetic at elevated temperatures. In our study, we analyze the magnetic excitation energy, along with the magnetic anisotropy to estimate the magnetic properties at finite temperatures.

Recently, machine learning (ML) has been combined with traditional methods (experiments and ab initio calculations) to advance rapid materials discovery^2,3,36,40–45^. ML models trained on a number of structures can predict the properties of a much larger set of materials. In particular, there is presently a growing interest in exploiting ML for discovery of magnetic materials^27,46^. ML studies of ferromagnetism in transition metal alloys have highlighted the importance of data analytics techniques to tackle problems in condensed matter physics^46^. In addition, the computational study of layered transition metal carbides and nitrides, known as MXenes, using high-throughput DFT relies on tuning the atomic composition of known MXenes to identify new ferromagnetic phases^32,47^. Further to this, recent work uses ML models to optimize the chemical composition of magnetic materials with three-dimensional crystal structures^27^. Therefore, it is conceivable that tuning the atomic composition could provide an additional degree of freedom in the search for stable 2D materials with interesting magnetic properties^48^. Even more compelling is the prospect of ML tools to assist in uncovering the physics underlying the stability and magnetism of 2D materials^49,50^. Specifically, ML methods can identify patterns in a high-dimensional space revealing relationships that could be otherwise missed^51,52^.

## Methodology

**Figure 1:**
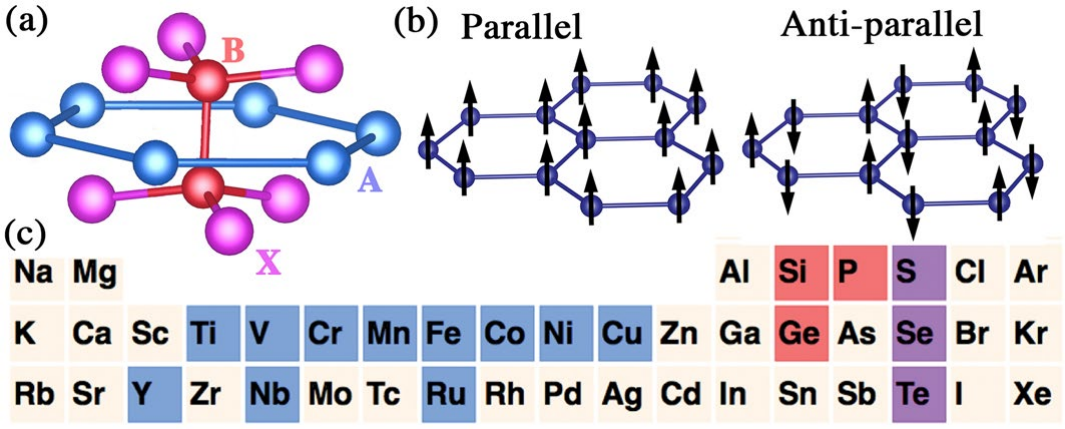
(**a**) Crystal structure of the $\hbox {A}_2\hbox {B}_2\hbox {X}_6$ lattice. (**b**) Magnetic orders considered in the A plane, labelled parallel and anti-parallel. (**c**) Elements used for substitution of A (blue), B (red) and X (magenta) sites.

In order to develop a path towards discovering 2D magnetic materials, we generate a database of structures based on monolayer $\hbox {Cr}_2\hbox {Ge}_2\hbox {Te}_6$ (Fig. 1a) using density functional theory (DFT) calculations with non-collinear spin and spin-orbit interactions included. The possible structures amount to a combinatorially large number of type $\hbox {A}_2\hbox {B}_2\hbox {X}_6$ ($\sim 10^4$) with different elements occupying the A, B and X sites. We select an initial subset of 198 structures due to computational constraints (and calculate additional structures at a later stage). We obtain the total energy, magnetic order, and magnetic moment of each structure. The ground-state properties were determined by examining the energies of the fully optimized structure with several spin configurations, including non-spin-polarized, parallel, and anti-parallel spin orientations at the A sites (Fig. 1b). The energy difference between parallel and anti-parallel spin configurations estimates an excited state property, the magnetic excitation energy. This is linked to the stability of magnetic order at finite temperatures. Using the Heisenberg model on a honeycomb lattice we extract the effective exchange energies *J* for the set of structures from their corresponding magnetic excitation energies^53^. The Curie temperatures can be estimated using analytical methods which involve *J* as well as the magneto-crystalline anisotropy (MCA)^5,54^. MCA is estimated by calculating the magnetic anisotropy energy^5,55^ (see Supplementary Information S1).

We then employ a set of materials descriptors which comprise easily attainable atomic properties, and are suitable for describing magnetic phenomena. We employ additional descriptors which are related to the formation energy^56^. The performance of descriptors in predicting the magnetic properties or thermodynamic stability sheds some light into the origin of these properties.

To create the database we use DFT calculations with the VASP code^57^. We used the GGA-PBE for the exchange-correlation functional^58^. The plane-wave energy cutoff was 300 eV for the initial set of calculations; this was increased to 450 eV at a later stage. The vacuum region was thicker than 20 Å. The atoms were fully relaxed until the force on each atom was smaller than 0.01 eV/Å. A $\Gamma$-centered $10\times 10 \times 1$*k*-point mesh was utilized.

We create the different structures by substituting one of two Cr atoms (A site) in the unit cell with a transition metal atom from the list: Ti, V, Cr, Mn, Fe, Co, Ni, Cu, Y, Nb, and Ru. In the two B sites we place combinations of Ge, Si, and P atoms, namely $\hbox {Ge}_2$, GeSi, GeP, $\hbox {Si}_2$, SiP, and $\hbox {P}_2$. The atoms at X sites were either S, Se, or Te, that is, $\hbox {S}_6$, $\hbox {Se}_6$, or $\hbox {Te}_6$. Figure 1c shows the choice of substitution atoms in the Periodic Table. An example of a structure created through this process is (CrTi)(SiGe)$\hbox {Te}_6$.

The careful choice of descriptors is essential for the success of any ML approach^59,60^. We use atomic properties data from the python mendeleev package 0.4.1^61^ to build descriptors for our ML models. We performed supervised learning with atomic properties data as inputs, with target properties the magnetic moment, the magnetic excitation energy and the formation energy. The choice of the set of descriptors for the magnetic properties was motivated by the Pauli exclusion principle, which gives rise to the exchange and super-exchange interactions. We also consider the magneto-crystalline anisotropy^62^ by building inter-atomic distances and electronic orbital information into our descriptors. With respect to the formation energy, the choice of descriptors was motivated, in part, by the extended Born-Haber model^56^, and include the dipole polarizability, the ionization energy and the atomic radius (see Supplementary Information S1 for a full list of atomic properties and descriptors used).

The data were randomly divided into a training set, a cross-validation set and a test set. Training data and cross-validation were typically 60% of the total data while test data comprised 40% of all the data. We employed the following ML models: kernel ridge regression, extra trees regression, support vector classification, and neural networks. Kernel ridge regression with a Gaussian kernel has been shown to be successful in several materials informatics studies. Extra trees regression allows us to determine the relative importance of features used in a successful model^63^. A support vector classifier was used to predict the low-energy magnetic order^64^. An analysis of hidden layers of the deep neural networks could allow us to identify patterns in 2D materials properties data, thereby guiding theoretical studies^50,52^.

## Results and discussion

### Magnetic properties

**Figure 2:**
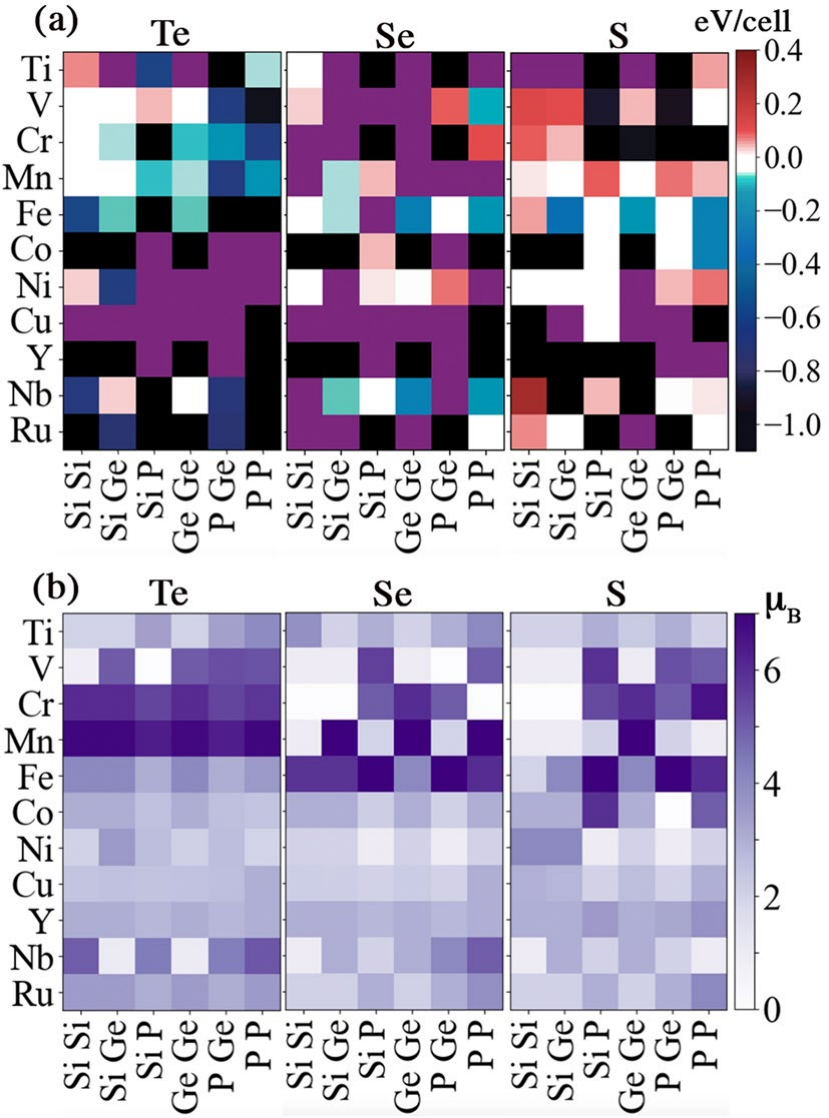
(**a**) Energy difference between parallel and anti-parallel spin configurations ($E_{\text {parallel}}-E_{\text {anti-parallel}}$ in $\hbox {eV}/\hbox {unit cell}$) of $\hbox {A}_2\hbox {B}_2\hbox {X}_6$ structures. Purple (black) represents a spin flip to the anti-parallel (parallel) configuration during DFT calculations, which makes the magnetic excitation energy unaccessible. (**b**) Magnetic moment per unit cell (in $\mu _B$) for each $\hbox {A}_2\hbox {B}_2\hbox {X}_6$ structure at the lowest energy spin configuration. The occupation of the two B sites is shown on the horizontal axis while that of one of the A site is shown on the vertical axis.

We find that the non-spin-polarized configuration has the highest energy for all the structures considered. That is, all structures prefer either parallel or anti-parallel ordering in the A plane. Figure 2a shows the energy difference of parallel and anti-parallel spin configurations. Negative (positive) energy difference means the parallel (anti-parallel) is more stable. We note that, because of the supercell size limit, we do not consider more complex spin configurations in this study. For example, the lowest-energy spin configuration of $\hbox {Cr}_2\hbox {Si}_2\hbox {Te}_6$ was reported to be zigzag anti-ferromagnetic type^53^. We find that for some structures, spin configurations initialized to one class may switch to the other during the calculation. That is, the higher energy spin configuration may not be readily constrained, causing difficulty in obtaining its energy. In this case, we expect a large magnitude for the magnetic excitation energy, although its value is unknown. In Fig. 2a we highlight the presence of spin flip in the calculations, where purple (black) color presents a spin flip to the anti-parallel (parallel) configuration during the DFT calculation.

This energy difference between parallel and anti-parallel spin configurations, namely, the magnetic excitation energy, is not only used to determine the magnetic order of a structure, it is also used to estimate the effective magnetic coupling strength *J* by the Heisenberg model with nearest-neighbor couplings. The magnetic excitation energy, together with the magnetic anisotropy energy, a key component of magnetism in two-dimensions^54^, are used to estimate the Curie temperature. We list a few examples of structures with Curie temperature higher than that of $\hbox {Cr}_2\hbox {Ge}_2\hbox {Te}_6$ in Table 1.

**Table 1:** Formation energy, magnetic moment per unit cell, magnetic excitation energy, and Curie temperature $T_\text {c}$ for monolayer $\hbox {Cr}_2\hbox {Ge}_2\hbox {Te}_6$ and structures with $T_\text {c}$ greater than that of $\hbox {Cr}_2\hbox {Ge}_2\hbox {Te}_6$.

Formula	$E_\text {f}$ [eV]	$\mu$ [$\mu _B$]	$\Delta E$ [eV]	$T_\text {c}\hbox { [K]}^a$
$\hbox {Cr}_2\hbox {Ge}_2\hbox {Te}_6$	− 1.74	6.19	− 0.08	84
$\hbox {Cr}_2\hbox {PGeTe}_6$	− 1.47	5.90	− 0.16	131
$\hbox {Cr}_2\hbox {SiPTe}_6$	− 1.72	5.92	− 0.14	126
$\hbox {CrFeSiGeSe}_6$	− 2.90	4.26	− 0.22	159
$\hbox {Cr}_2\hbox {SiPSe}_6$	− 3.95	5.73	− 0.13	237
$\hbox {CrCoSiPS}_6$	− 3.98	3.82	− 0.23	129
$\hbox {CrFeP}_2\hbox {S}_6$	− 2.94	5.63	− 0.28	167
$\hbox {CrFeSiPS}_6$	− 3.97	6.28	− 0.06	142

${}^a$The Curie temperature was estimated using the procedure in Ref. ^54^.

Total magnetic moments for the lowest energy spin configuration of each structure are presented in Fig. 2b. There are 14 structures that have magnetic moments higher than that of $\hbox {Cr}_2\hbox {Ge}_2\hbox {Te}_6$. Examples of these structures include $\hbox {(CrMn)Si}_2\hbox {Te}_6$, $\hbox {(CrFe)(SiP)Se}_6$, and $\hbox {(CrFe)(GeP)S}_6$, which exhibit magnetic moments up to $7\,\mu _B$ per unit cell. We find that only atoms in the A sites show finite magnetic moments, while the moments in the B and X sites are small. Distinct patterns for regions of high and low magnetic moments are observed for X = Te, Se and S in Fig. 2b. Structures created by substituting non-magnetic atoms at the A site, such as Cu, have small variations in their relatively small magnetic moments, as seen in the rows of Fig. 2b. However, substitutions of magnetic atoms, such as Mn, result in a set of structures with a large variation in the magnetic moment, with a much larger upper limit to the range of values observed.

Both the magnetic order and magnetic moment are sensitive to the occupancy of B and X sites, even though the atoms in these sites have negligible contribution to the overall magnetic moment. Atoms in the X sites strongly mediate the magnetic coupling between neighboring A sites^53^. Atoms at the B sites can affect the relative positions of A and X sites. Direct exchange between first nearest neighbor A sites competes with super-exchange interactions mediated by the $\textit{p}$-orbitals at the X sites. The ground state magnetic order is determined by the interplay between first, second and third nearest neighbor interactions. Changing the identity of one of the A, B or X sites affects the interplay between the direct exchange and super-exchange interactions. Recent work has shown that applying strain to the $\hbox {Cr}_2\hbox {Si}_2\hbox {Te}_6$ lattice tunes the first nearest neighbor interaction, resulting in a change in the magnetic ground state from zig-zag antiferromagnetic to ferromagnetic^53^. Our work demonstrates that tuning the composition of the $\hbox {A}_2\hbox {B}_2\hbox {X}_6$ lattice can have an equivalent effect. For instance, whereas X=Te structures show more parallel ($\bar{{\bar{P}}}$) than anti-parallel (anti-$\bar{{\bar{P}}}$) spin-configurations with lower energy, there is a clear change when X $=$ Se or S. As X moves up the periodic table, there are increasingly more regions of anti-parallel spin configuration, as well as regions in which $\bar{{\bar{P}}}$ and anti-$\bar{{\bar{P}}}$ are degenerate. In particular, we find that the distance between nearest neighbor A and X sites, as well as two adjacent X sites is linked to the magnitude of the magnetic moment (see Supplementary Information S1 for details).

**Figure 3:**
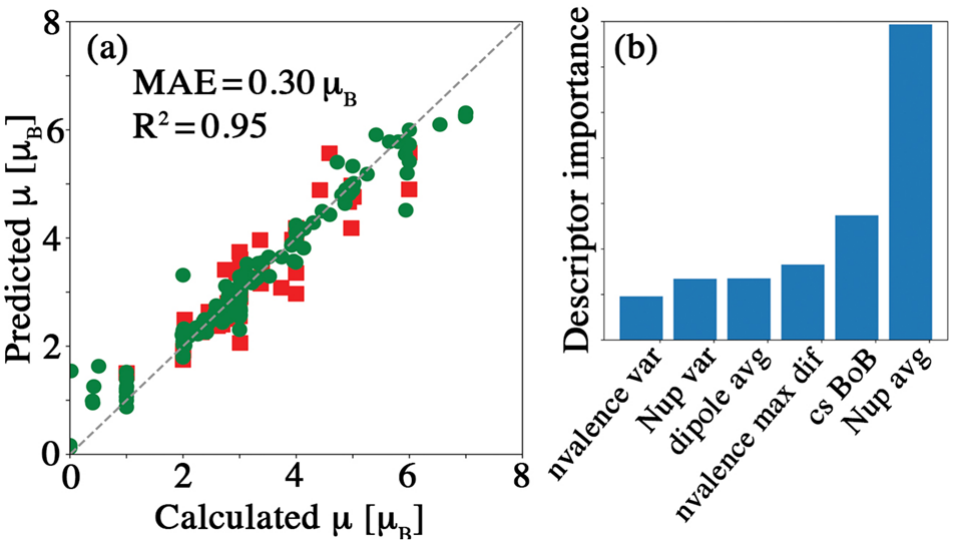
ML predictions of magnetic moments of $\hbox {A}_2\hbox {B}_2\hbox {X}_6$ structures. (**a**) Extra trees model performance for the magnetic moment (in $\mu _B$) prediction. A subset of structures for X = Te are displayed. The red squares indicate the test data, the green circles show the training data. (**b**) Top six descriptors for the extra trees prediction of the magnetic moment. The size of the bar indicates relative descriptor importance (see text for details).

We use extra trees regression^63^ to approximate the relationship between the total magnetic moment and a set of descriptors designed for magnetic property prediction (see Supplementary information S1). Training and test data are considered for the X = Te, Se, and S structures individually. The model performance for X = Te, using a data set with size $N = 262$ (see Supplementary Information S1), is shown in Fig. 3a. We find reasonable prediction performance for X = Te that deteriorates for X = Se and is even worse for X = S. This suggests that our model, along with the set of descriptors used to predict X = Te structures, is not easily optimized to include X=Se and S structures. This could arise due to the fact that there are more structures that have degenerate $\bar{{\bar{P}}}$ and anti-$\bar{{\bar{P}}}$ spin configurations for X = Se and S than for X = Te. Furthermore, the magnetic moment for X = Se and S structures have larger variations across B sites when compared to X = Te structures. These variations likely pose a learning challenge for statistical models. Subgroup discovery^65^ implies that the identity of the X site strongly affects the magnetic properties of the structures. We included a modified Bag of Bonds descriptor^66^ to capture the orbital overlap between adjacent sites. The model performs poorly for X = Se and S, perhaps because of missing second and third nearest-neighbor interactions in the descriptor that are important in determining magnetic couplings.

**Figure 4:**
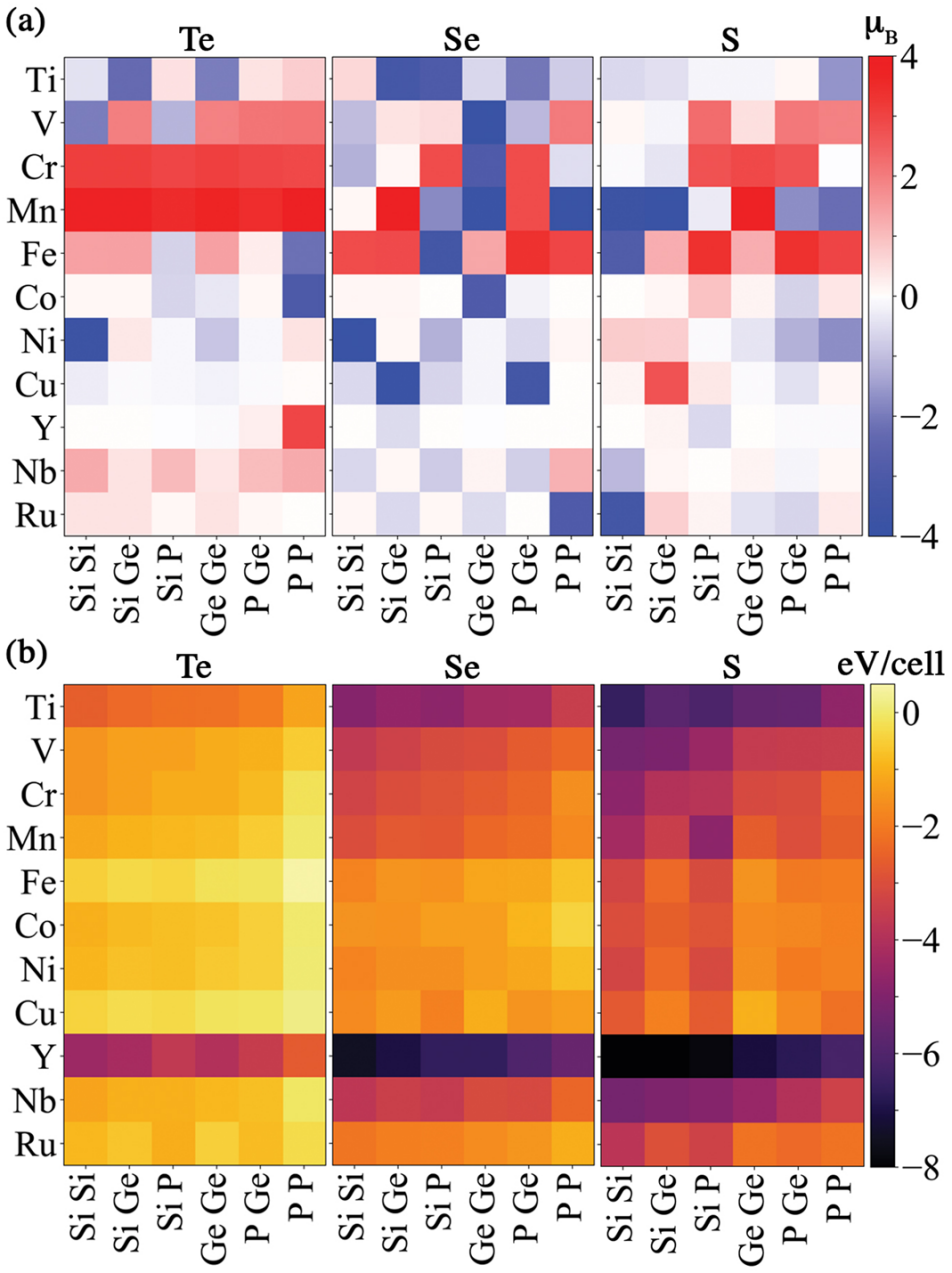
(**a**) Local magnetic moment of the transition metal A site, $\hbox {A}_{\text {TM}}$ (in $\mu _{\text {B}}$). (**b**) Formation energy (in eV/cell) for $\hbox {A}_2\hbox {B}_2\hbox {X}_6$ structures at the lowest energy spin configuration. Conventions are the same as in Fig. 2.

Determining which descriptors are most important for making good predictions of a property can be exploited for knowledge discovery, especially when a large number of descriptors are available but their relationships with the target property are not known^67^. Figure 3b shows the descriptor importances^64^ as derived from extra trees regression. It shows that the following are among the top six descriptors in the set examined: (i) the ‘*average number and variance of spin up electrons*’ (“Nup avg” and “Nup var” in Fig. 3b), which are linked to the atomic magnetic moments, (ii) the ‘*chemical space value*’ (“cs BoB”, a modified Bag of Bonds descriptor^66^, see Supplementary Information S1), (iii) the ‘*maximum difference and variance of valence electron number*’ (“nvalence max dif” and “nvalence var”), and (iv) the ‘*average dipole polarizability*’ (“dipole avg”). The magnetic moment per unit cell is a function of the magnetic moments of the individual atoms in the unit cell. However, determining the exact value and the orientation of the $\mu$ localized at each site is not trivial. We examine the local magnetic moments at the A sites to determine how the magnetic moment per unit cell is constructed. The local magnetic moment at the A sites ($\hbox {A}_{\text {Cr}}$ and $\hbox {A}_{\text {TM}}$) can be different from the atomic dipole magnetic moment of the corresponding element. For instance, while the atomic magnetic moment of $\hbox {Cr}^{3+}$ is $3~\mu _B$, the local magnetic moment at $\hbox {A}_{\text {Cr}}$ fluctuates from 2.7 to $3.2~\mu _B$. Fig. 4a shows the local magnetic moment at $\hbox {A}_{\text {TM}}$. The atomic magnetic moment can be roughly considered as an upper limit of the magnetic moment of the corresponding lattice site in a compound. It is also linked to the magnetic moment per unit cell. The model prediction error for the magnetic moment per unit cell is increased by only 1% when the atomic magnetic moments are excluded from the set of descriptors. This suggests that there exists redundancy in the descriptor space. For predicting properties in which the physics involved is sophisticated, such redundancy seems inevitable.

Furthermore, we use the magnetic excitation energy data in Fig. 2a to train a support vector classification model, to predict the ground-state magnetic order of $\hbox {A}_2\hbox {B}_2\hbox {X}_6$ structures. The ground state is FM (AFM) if the magnetic excitation energy is negative (positive). We achieve an $82\%$ success rate for the prediction of ferromagnetic order. Antiferromagnetic order prediction has an $80\%$ success rate (see Supplementary Information S1). Attempts to use a regression model to predict the amplitude of the magnetic excitation energy are not successful, perhaps due to missing physics in the descriptor space or insufficient quantities of training data to learn the sophisticated physics.

### Formation energy

In addition to identifying structures with specific magnetic properties, the ability to screen for chemical stability is also important. DFT-calculated formation energies (for the lowest energy spin configuration) are shown in Fig. 4b. We note that the formation energy in this work is referenced to the corresponding elemental phases. Since the errors from DFT are usually inconsistent between pure elemental phases and compounds, there are potential errors in the absolute values of formation energy. This can be improved in a future work by utilizing fitted elemental-phase reference energies^68^. Presently, we do not use the energies of the competing compound phases to calculate formation energies, due to lack of information about the competing phases in the synthesis (see Supplementary information S1) .

Structures comprising certain elements, such as Y, decrease the formation energy considerably in comparison to those without it. Certain transition metals, such as Cu, tend to destabilize the $\hbox {(CrA)B}_2\hbox {X}_6$ structures. The formation energy, $E_f$ becomes less negative as the substituted atom at the A site goes from the left to the right of the first and second row of transition metal elements in the Periodic Table. This is linked to the filling of the *d*-orbital, where elements with a filled *d*-orbital do not form chemical bonds with other elements. Varying the composition at the B site does not appear to have a strong impact on the formation energy (see Supplementary Information, Fig. S1). Changing the X site from Te to Se and then S results in the overall trend of decreasing formation energy.

**Figure 5:**
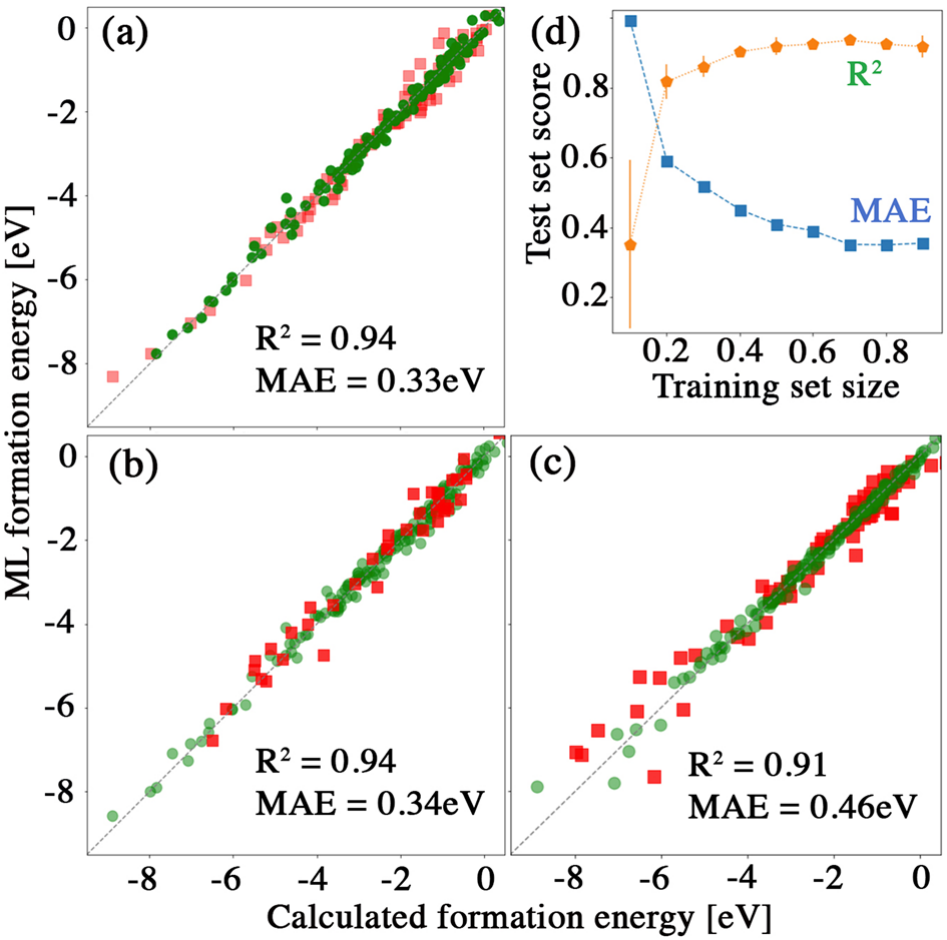
Formation energy prediction performance of (**a**) kernel ridge regression, (**b**) deep neural network regression and (**c**) extra trees regression. Red squares are test data and green circles training data. (**d**) Performance of the extra trees regression model on the test data as the training set size increases, in terms of the $\hbox {R}^2$ and mean absolute error (MAE) scores.

To exploit the trends in the formation energy data, we use statistical models to predict the formation energy and to infer structure-property relationships. We find that some descriptors, such as the atomic dipole polarizability, are strongly correlated with the formation energy, and are therefore important in generating good ML predictions. Since useful descriptors are not always revealed in an analysis of the Pearson correlation coefficient^67^, we consider other methods to learn descriptor importances such as the extra trees model^64^. Using the ML models to predict the formation energy of $\hbox {A}_2\hbox {B}_2\hbox {X}_6$ structures permits the quick calculation of the formation energy for a large set of compounds. Whereas DFT calculations of $10^4$ structures could require much more than 1 million CPU hours, the ML prediction takes a few seconds. Figure 5a shows the prediction performance for kernel ridge regression using a Gaussian kernel. Figure 5b shows the performance of a neural network (The neural network is implemented by tensorflow^69^. It is comprised of 3 hidden layers with sizes 10, 30 and 10 units) while Fig. 5c shows the performance of the extra forests regression. Both training set and test set results are displayed, as well as the test scores for kernel ridge regression, extra trees regression, and neural network regression.

**Figure 6:**
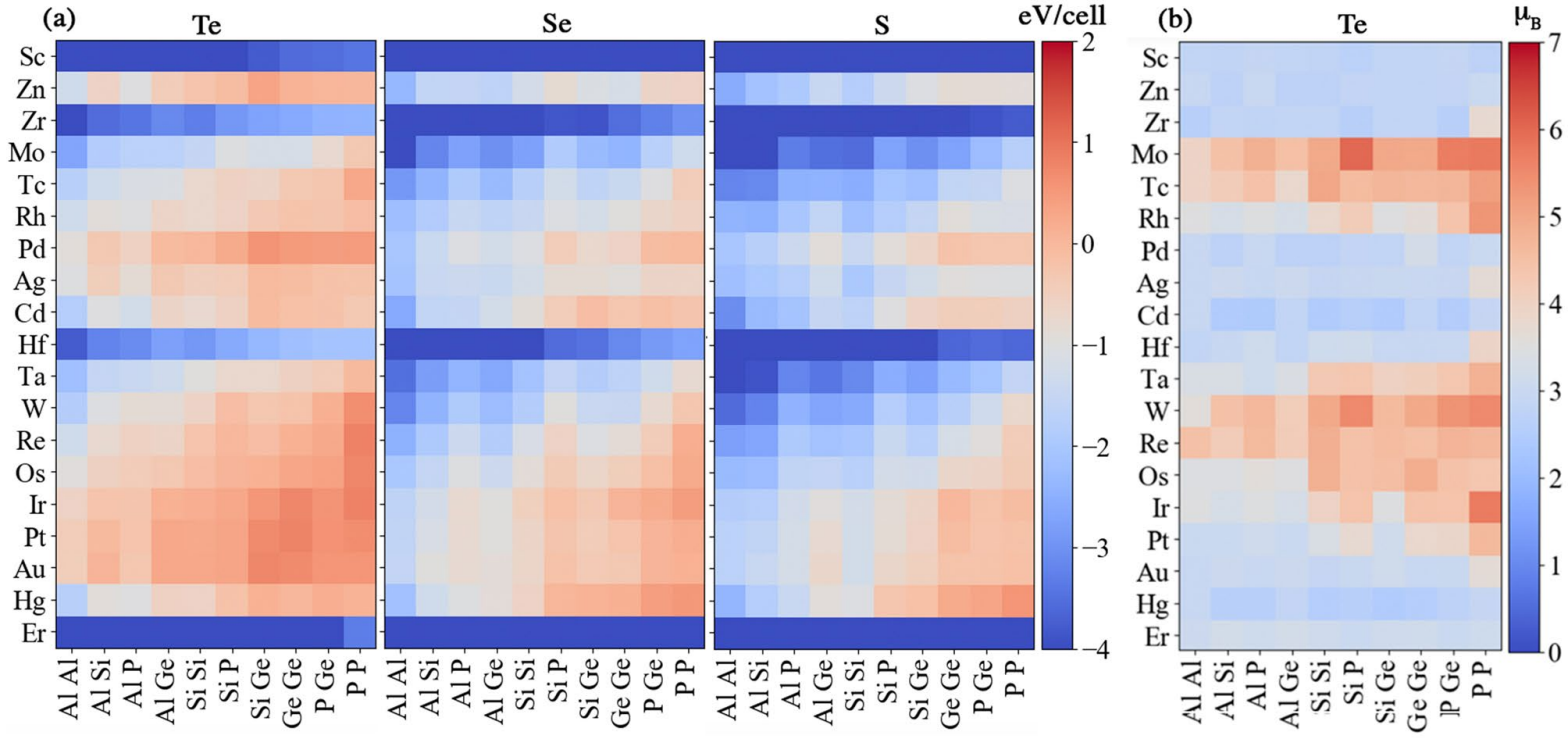
(**a**) ML predicted formation energies (in eV/cell) for a wide range of substitutions that were not included in the DFT data set covering 4223 new structures (570 are shown here). (**b**) The first-step ML predicted magnetic moments (in $\mu _B$) for a wide range of substitutions that were not included in the DFT data set covering 4223 new structures (190 are shown here for X=Te). Conventions same as in Fig. 2.

Further analysis (see Supplementary Information S1) shows that the ‘*variance in the ionization energy of atoms*’ and the ‘*average number of valence electrons*’ are the two most important descriptors in the set examined. This demonstrates a link between the formation energy and the atomic ionization energy, emanating from the increased atomic ionizability which produces stronger chemical bonding. In addition, the number of valence electrons is linked to the number of electrons available for bonding. For instance, substitutions by atoms with a filled outer orbital shell will create less stable bonds, leading to chemical instability. The ability of our models to generalize is demonstrated by the high scores on the test data. We further examined how the test set performance varies with the training set size. Figure 5d shows test scores as a function of training set size using extra trees regression. The test score reaches a plateau at about a training set size of 40%, with test score ($\hbox {R}^2$) as high as 0.91.

### High-throughput screening using ML models

We can use our trained ML models to make predictions on a wide range of structures not included in the original DFT data set. Thus far, we have used our ML models to estimate the formation energy for an additional 4,223 $\hbox {A}_2\hbox {B}_2\hbox {X}_6$ structures, constructed as follows: (i) For A site substitutions, we considered transition metals not used in the DFT dataset. (ii) We included Al, Sn and Pb in the set of atomic substitutions for B sites (not shown). (iii) For the X sites, we added O to our previous choice of S, Se and Te. The resulting predictions, partly shown in Fig. 6a, provide a means to quickly screen a large data set of structures for chemical stability. For instance, our ML predictions suggest that structures based on Er, Ta, Hf, Mo, Zr, and Sc in the A site and Al in the B site are likely to be stable and thus good candidates for further exploration.

We use a two-step process to find materials with high magnetic moments. In the first stage, a regression model trained with a small data set size ($N = 66$) was used to estimate the magnetic moment. The magnetic moment predictions are shown in Fig. 6b. From the results of the ML predictions we select structures with formation energies below $-1.0\hbox { eV}$ and magnetic moments above $5~\mu _B$ (for X=Te only). From the 4223 predictions, we obtained 40 that satisfied our constraints. 15 of these were randomly selected for DFT verification. The 15 structures have relatively low formation energies and high magnetic moments, but only 5 of them fulfill the criteria with hard cutoffs (formation energy lower than $-1.0\hbox { eV}$ and magnetic moment higher than $4.5\,\mu _B$).

The second round of model training included the additional 15 structures calculated by DFT. This improved model was then used to predict the magnetic moment. Surprisingly, all the candidate structures predicted by the new model were verified to meet the criteria by DFT. They are $\hbox {(CrMo)Si}_2\hbox {Te}_6$ ($E_\text {f} = -1.32\hbox { eV}$, $\mu = 6.00\,\mu _B$), $\hbox {(CrW)Si}_2\hbox {Te}_6$ ($E_\text {f} = -1.11\hbox { eV}$, $\mu = 5.89 \mu _B$), and $\hbox {(CrMo)(SiP)Te}_6$ ($E_\text {f} = -1.10\hbox { eV}$, $\mu = 5.01\,\mu _B$). This shows that the model can be substantially improved by feeding accurate DFT data from structures that are close to the phase space linked to desirable properties. The first step narrows down the target region where candidate systems are more likely to appear. By sampling the target region using DFT and feeding data to the training in the second step, the resulting model learns to distinguish the desirable structures more accurately. This two-step or iterative method, analogous to active learning^70^, is capable of building accurate models for materials discovery using limited quantities of training data.

We computed phonon spectra for some of the above promising structures using the open-source software package phonopy^71^ in the frozen phonon method. We relaxed our unit cell to very high precision (within 0.0001 eV/A) with cutoff energies ranging from 400 to 500 eV with a substantially finer mesh to model the augmentation charges around ions, with an augmentation energy cutoff (ENAUG in VASP) of 2000 eV. We found that the candidate compounds $\hbox {CrMo}\hbox {Si}_2\hbox {Te}_6$, $\hbox {CrW}\hbox {Si}_2\hbox {Te}_6$, and $\hbox {CrMnSi}_2\hbox {Te}_6$ evidenced dynamical stability (see Supplementary information S1 for phonon spectra and further discussion).

## Discussions and conclusion

We presented evidence that the magnetic properties of $\hbox {A}_2\hbox {B}_2\hbox {X}_6$ monolayer structures can be tuned by making atomic substitutions at A, B, and X sites. This provides a non-traditional framework for investigating the microscopic origin of magnetic order of 2D layered materials and could lead to insights into magnetism in systems of reduced dimension^23,24^. Our work represents a path toward tailoring magnetic properties of materials for applications in spintronics and data storage^72^. We showed that ML methods are promising tools for predicting the magnetic properties of 2D magnetic materials. In particular, our data-driven approach highlights the importance of the X site in determining the magnetic order of the structure. Changing the composition of the $\hbox {A}_2\hbox {B}_2\hbox {X}_6$ structure alters the inter-atomic distances and the identity of electronic orbitals. This impacts the interplay between first, second and third nearest neighbor exchange interactions, which determines the magnetic order.

One goal of this work was to find magnetic 2D materials that are also thermodynamically stable. ML models were trained to predict chemical stability that allow the rapid screening of a large number of possible structures. We showed that the chemical stability of $\hbox {A}_2\hbox {B}_2\hbox {X}_6$ structures based on $\hbox {Cr}_2\hbox {Ge}_2\hbox {Te}_6$ can be tuned by making atomic substitutions. Examples of structures that satisfy both magnetic moment and formation energy requirements include the following: $\hbox {(CrMo)Si}_2\hbox {Te}_6$, $\hbox {(CrW)Si}_2\hbox {Te}_6$, and $\hbox {(CrMo)(SiP)Te}_6$, which are not included in our original DFT database. In addition, we found structures in our set of DFT calculations that also satisfied our requirements:

$$\hbox {(CrMn)Si}_2\hbox {Te}_6$$ ($$E_\text {f} = -1.77\hbox { eV}$$, $$\mu = 7.02$$$$\mu _B$$), $$\hbox {(CrMn)Ge}_2\hbox {Se}_6$$ ($$E_\text {f} = -3.24\hbox { eV}$$, $$\mu = 7.00\,\mu _B$$), $$\hbox {(CrFe)(SiP)S}_6$$ ($$E_\text {f} = -3.97\hbox { eV}$$, $$\mu = 6.99\,\mu _B$$), $$\hbox {(CrFe)(GeP)Se}_6$$ ($$E_\text {f} = -2.28\hbox { eV}$$, $$\mu =6.99 \mu _B$$), and $$\hbox {Cr}_2\hbox {Ge}_2\hbox {Se}_6$$ ($$E_\text {f}= -3.67\hbox { eV}$$, $$\mu = 6.02\,\mu _B$$). Furthermore, we included temperature effects by exploiting the magnetic excitation energy and the magnetic anisotropy energy to estimate the Curie temperature. We identified several structures with magnetic excitation energy much greater than that of $$\hbox {Cr}_2\hbox {Ge}_2\hbox {Te}_6$$, which corresponds to a higher Curie temperature. In Table 1 we show seven $$\hbox {A}_2\hbox {B}_2\hbox {X}_6$$ structures which may have Curie temperatures above that of $$\hbox {Cr}_2\hbox {Ge}_2\hbox {Te}_6$$. $$\hbox {(CrTc)(SiSn)Te}_6$$ and $$\hbox {(CrTc)Sn}_2\hbox {Te}_6$$ were also ML recommended structures which we excluded because of the radioactive elements they contain. The recommendations we generated can then be subjected to additional screening with more computationally expensive tests for chemical stability^73,74^, such as calculations of the dynamic stability^75,76^. The most promising results will serve as viable options for materials synthesis and experimental verification.

Subsequent to generating ML predictions of vdW magnets of the form $\hbox {A}_2\hbox {B}_2\hbox {X}_6$, we sought to verify the chemical stability and magnetic properties of our candidate structures by performing a literature search of each candidate. Our ML guided literature review revealed that many materials of the type $\hbox {A}_2\hbox {B}_2\hbox {X}_6$ have been synthesized and their magnetic properties characterized^77,78^. Reference^77^ presents a review of experimental studies done on bulk crystals of transition metal phosphorous trisulfides. There are over 10 structures reported to have been synthesized which overlap with those predicted in this study. Since our study is restricted to monolayers, we cannot directly compare our results with experiments described in Ref.^77^. However, the review highlights that many structures of the form $\hbox {A}_2\hbox {B}_2\hbox {X}_6$ exist in nature.

A second review article^78^ highlights experimental studies of bulk layered metal thio(seleno) phosphates, $\hbox {APX}_3$. A is a transition metal and X = (S, Se). For instance, $\hbox {CuAP}_2\hbox {Se}_6$ (A=In, Cr) compounds have been synthesized and studied. $\hbox {CuCrP}_2\hbox {Se}_6$ is one of the structures studied in our work. To the best of our knowledge none of the layered materials reported in Ref.^78^ have been thinned down to the monolayer. Nevertheless, these findings suggest that our approach provides a successful framework for the targeted investigation of monolayers of this class of material.

This work provides the impetus for further exploration of structures with other architectures not considered here, that is, with more complex atomic substitutions beyond 1 in 2 replacement of Cr atoms at the A site. We estimate a total number of at least $3\times 10^4$ structures of the $\hbox {A}_2\hbox {B}_2\hbox {X}_6$ type described in Fig. 1. A computationally efficient estimation of the magnetic properties and formation energy is required to quickly explore this vast chemical space. We have already transferred our materials informatics framework to a study of a different family of crystal structures, the transition metal dichalcogenides (TMDs). We successfully predicted the formation energies of TMDs using machine learning models trained on a database of DFT calculations^36^. The detailed results will be presented in a separate work. We expect the ML methods explored here, with proper modification, to allow an efficient exploration of other families of 2D magnets, such as $\hbox {CrI}_3$, CrOCl and $\hbox {Fe}_3\hbox {GeTe}_2$^23,28,79^.

During the review process we came across a similar study which also uses machine learning to predict the properties of 2D magnetic materials^80^. This work exploited data from the C2DB^5^ database and used different descriptors from those presented in this study.

## Supplementary information

Supplementary material 1

## Data Availability

The results of these DFT calculations will be used to build a database of monolayer 2D materials which will be publicly available to the scientific community. See the Supplementary Information for details.
