# Supplementary material for: Data-driven studies of magnetic two-dimensional materials
Source: Sci Rep. 2020 Sep 25;10:15795. doi: 10.1038/s41598-020-72811-z (PMC7519137; doi:10.1038/s41598-020-72811-z)
Supplement: Supplementary file 1 — Supplementary material 1 [file 41598_2020_72811_MOESM1_ESM.pdf]

Supplementary information for data-driven studies of magnetic two-dimensional materials

Trevor David Rhone[1,*], Wei Chen[1], Shaan Desai[1], Steven B. Torrisi[1], Daniel T. Larson[1], Amir Yacoby[1], and Efthimios Kaxiras[1,2]

[1]Department of Physics, Harvard University, Cambridge, Massachusetts 02138, USA
[2]School of Engineering and Applied Sciences, Harvard University, Cambridge, Massachusetts 02138, USA
[*]trr715@g.harvard.edu

1 The data set

1.1 Choice of chemical substitutions

We determined the elemental substitutions for the $CrAB_2X_6$ lattice after considering a set of well studied layered transition metal trichalcogenides[1;2;3]. Ref. 3 performed DFT calculations on monolayer $Cr_2X_2Te_6$ (where X=Si, Ge) and $Mn_2P_2X_6$ (where X=S, Se). Guided by these known layered magnetic bulk materials and DFT monolayer calculations, we chose $Cr_2Ge_2Te_6$ to be our archetype structure ($A_2B_2X_6$) from which we would make substitutions at the A, B and X sites. We kept one of two Cr atoms at the A sites fixed. The transition metal substitutions at the A site were chosen to explore most of the column numbers in the first row of the transition metals. We also randomly chose three elements in the second row to allow greater variety in the data. Though we would have liked to include additional transition metals in the first round of calculations, our computational resources were limited. We chose elements for the B sites to be Si, Ge and P due to the elements present in the experimentally reported bulk structures. X sites were chosen to be the chalcogens – Te, Se, S. Because of limited computational resources our initial search was limited to 198 structures. However, at a later stage, additional calculations were performed which explored other elements in the second and third row of the transition metal elements for A site substitutions. Also, Sn was added to the list of elements for B site substitutions.

1.2 Data availability

The results of the density functional theory calculations performed in this study are available for download at the Materials Cloud website (https://www.materialscloud.org) and can be access directly using the following link: https://doi.org/10.24435/materialscloud:2019.0020/v1

2 Data visualization of $A_2B_2X_6$ structures

The data generated from DFT calculations can be visualized in order to learn how the magnetic moment and formation energy vary across the set of $A_2B_2X_6$ structures. For instance, we can examine how substitutions at one of the A sites with a given transition metal (TM) impacts the formation energy of a set of structures. Fig. S1(a) shows a histogram of formation energies for various TM substitutions. The histograms indicate that TM substitution can substantially affect the formation energy. For example, structures containing Cu tend to have a formation energy close to zero (i.e. they are chemically unstable).

Fig. S1(b) shows the distribution of magnetic moments for A site substitutions with TM atoms (i.e. one of the two A sites is substituted with a TM atom). Different TM atoms have varying impact on the total magnetic moment per unit cell. We argue in the main text that the magnetic ordering of the structures is affected by the distance between adjacent X sites and from an X site to an A site. Fig. S2(b) and (c) shows how the distance between an A and X site (e.g. a_1x_1, a_1x_2) and between adjacent X sites (e.g. x_1x_2) is linked to the magnetic moment. We performed principal component analysis (PCA)[4] on a set of features comprising inter-site distances. These include the distance between the following pairs of sites: a_1a_2, a_1b_1, a_1b_2, a_1x_1, a_1x_2, a_2b_1, a_2b_2, a_2x_1, a_2x_2, b_1x_1, b_1x_2 and x_1x_2. Fig. S2(d) shows that the magnetic moment per unit cell varies with the first principal component. Fig. S2(e) shows how the magnetic moment (see color scale) varies with the first and third principal components - clustering of regions of large μ is clearly shown. This demonstrates a relationship between the magnetic moment and the relative positions of the sites - in particular, the X and A sites.

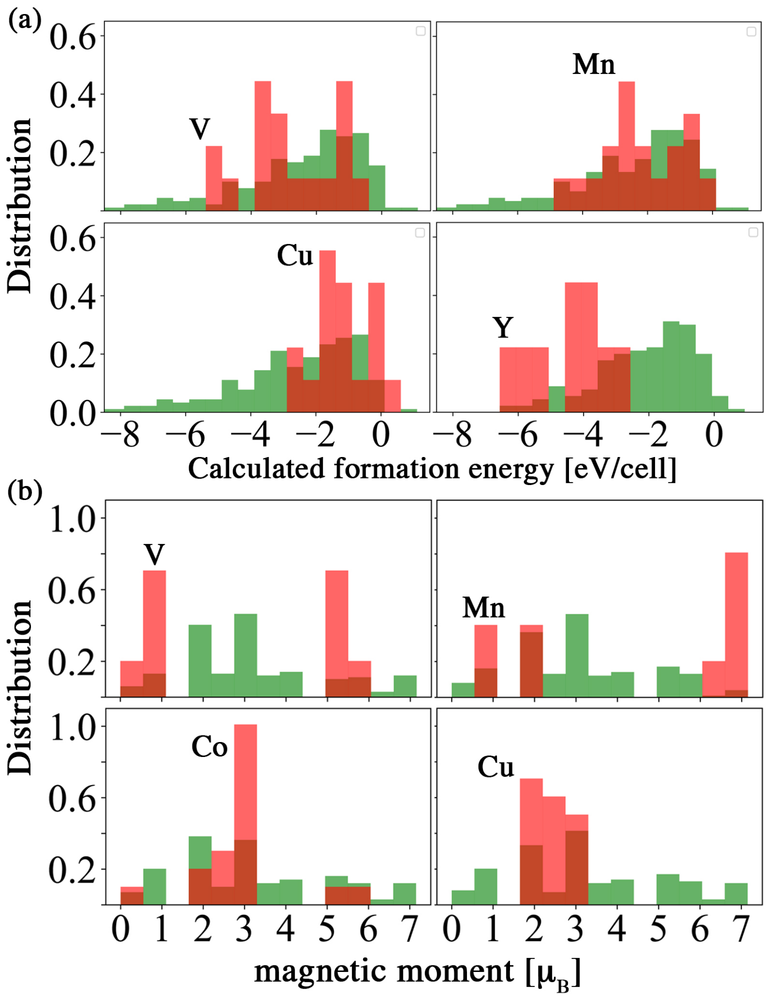

Figure 1. (a) Distributions of the DFT-calculated formation energies for composites with (red) and without (green) a specific substitution atom in one of the A sites. (b) Distributions of the magnetic moment for a set of composites with (red) and without (green) a specific substitution transition metal. The target atomic species (Y, Mn, V, and Cu) is specified in each panel.

3 Principal component analysis

Principal component analysis (PCA) is a data analysis tool, often used for dimensionality reduction[4]. It can be exploited for data visualization, where a high-dimensional space of descriptors is reduced to two-dimensions which can then be plotted (see Fig. S2). In addition, PCA is used alongside regression to create more robust models which avoid overfitting. PCA works by defining an orthogonal set of basis vectors which are ranked in terms of the variance in the data. That is, the first principal component represents a linear combination of the original set of descriptors that captures the most variance in the data. The coefficients of the vector represent the contribution from the original set of descriptors. Since the target property is not used to determine the principal components, there will not necessarily be a correlation between the target property and the principal components. However, it can be useful to determine which descriptors are important for predicting a target property. This is discussed in the next section.

4 Materials descriptors

The choice of descriptor plays a crucial role in the success of the machine learning prediction, and the interpretability of the descriptors allows new insights. Descriptor D_i for the ith structure was constructed from atomic properties as follows:

$$D_i = f(p_A, p_{A'}, p_B, p_{B'}, p_X) \tag{1}$$

where D_i is a function of atomic properties p of elements A, A′, B, B′ and X of the ith structure. The function $f(p_A, p_{A'}, p_B, p_{B'}, p_X)$ describes the statistics of a set of atomic properties. This can be the arithmetic mean of all the atomic properties (e.g. mean(p_A,

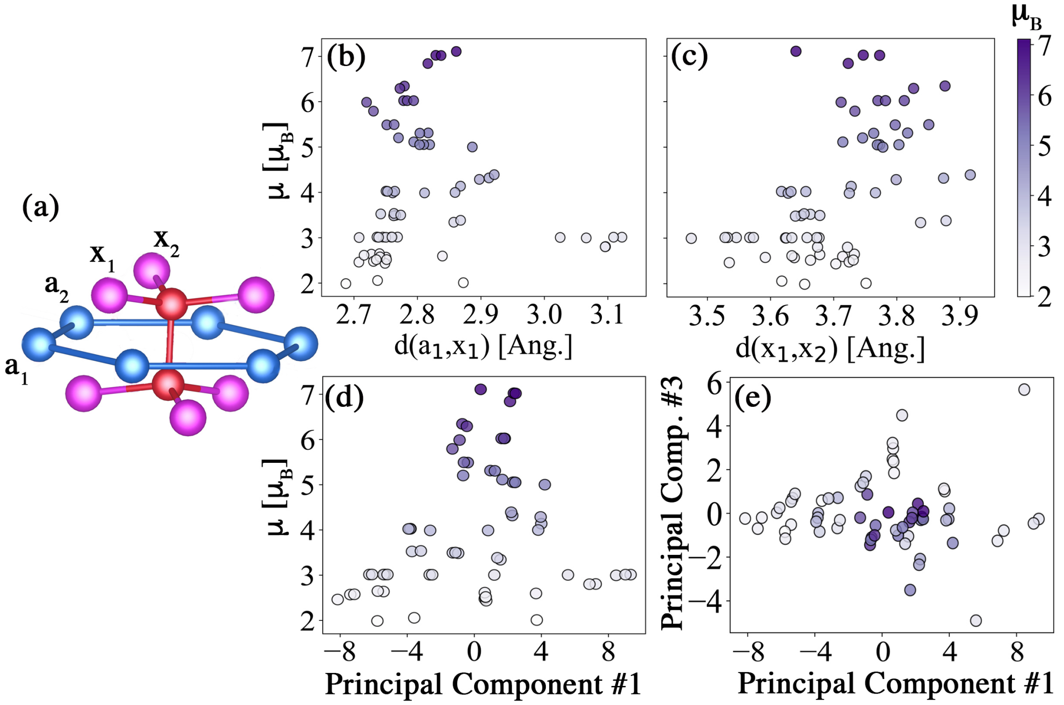

Figure 2. (a) $A_2B_2X_6$ structure with sites labelled. (b) Distance between adjacent a_1 and x_1 sites with respect to the magnetic moment. (c) Distance between adjacent X sites with respect to the magnetic moment. (d) and (e) show the relationship between the magnetic moment per unit cell and the first and third principal components.

Table 1. Descriptor importances for magnetic moment prediction. The top 12 out of 50 are shown.

Rank	Descriptor	Rank	Descriptor
1	number of spin up electrons (mean)	7	number of valence electrons (mean)
2	chemical space BoB	8	dipole polarizability (var. of differences)
3	number of valence electrons (max. difference)	9	atomic radius (mean)
4	dipole polarizability (mean)	10	covalent radius (var. of differences)
5	number of spin up electrons (var.)	11	dipole polarizability (max. difference)
6	number of valence electrons (var.)	12	hardness (mean)

$p_{A'}$, p_B, $p_{B'}$, p_X)), the mean of the differences of adjacent pairs, the sum, the sum of the differences of adjacent pairs, the variance, the variance of the differences of adjacent pairs (i.e. var($p_A - p_{A'}, p_A - p_B, p_A - p_{B'}, p_A - p_X, p_{A'} - p_B, p_{A'} - p_{B'}, p_{A'} - p_X$)) or the maximum in a set of differences of adjacent pairs, max($p_A - p_{A'}, p_A - p_B, p_A - p_{B'}, p_A - p_X, p_{A'} - p_B, p_{A'} - p_{B'}, p_{A'} - p_X$). This approach for constructing descriptors is similar to that used by Ghiringhelli et al.[5].

The atomic properties, p considered were the following: dipole polarizability, ionization energy, atomic radius, number of valence electrons, number of electrons, electronegativity, atomic volume, van der Waals radius, covalent radius, number of spin up electrons and chemical hardness[6;7] as defined by the python package mendeleev (Version 0.4.1).

We attempted to improve the molecular representation of the ABX_3 structures by designing a descriptor to contain information on the direct exchange interaction. We modified the Bag of Bonds (BoB) descriptor[8] by incorporating the relationship between the exchange coupling and the interatomic distance[9]. That is, we replace the Coulomb Kernel[10] in the BoB with an estimate of the term parameterizing the Bethe-Slater curve, the interatomic distance normalized by the radius of the incompletely filled shell: $(J_i + J_j)(N_i^{up} + N_j^{up})$ for pairs of i and j atoms in a structure. J is the product of the atomic dipole polarizability and the covalent radius, N^{up} is the number of unpaired electrons.

4.1 Descriptor importance

The extra trees regression model can rank the descriptors used in a successful model in order of importance[4]. The top twelve descriptors (in decreasing order of importance out of a total of 50) for the magnetic moment prediction are shown in Table 1,

Table 2. Descriptors importances for formation energy prediction. The top 12 out of 50 are shown.

Rank	Descriptor	Rank	Descriptor
1	ionization energy (var.)	7	atomic volume (sum of differences)
2	# valence electrons (mean)	8	atomic volume (mean)
3	dipole polarizability (max. difference)	9	covalent radius (max. difference)
4	dipole polarizability (var.)	10	atomic radius (var. of differences)
5	atomic volume (max. difference)	11	atomic radius (sum of differences)
6	atomic radius (max. difference)	12	# of spin up electrons (mean)

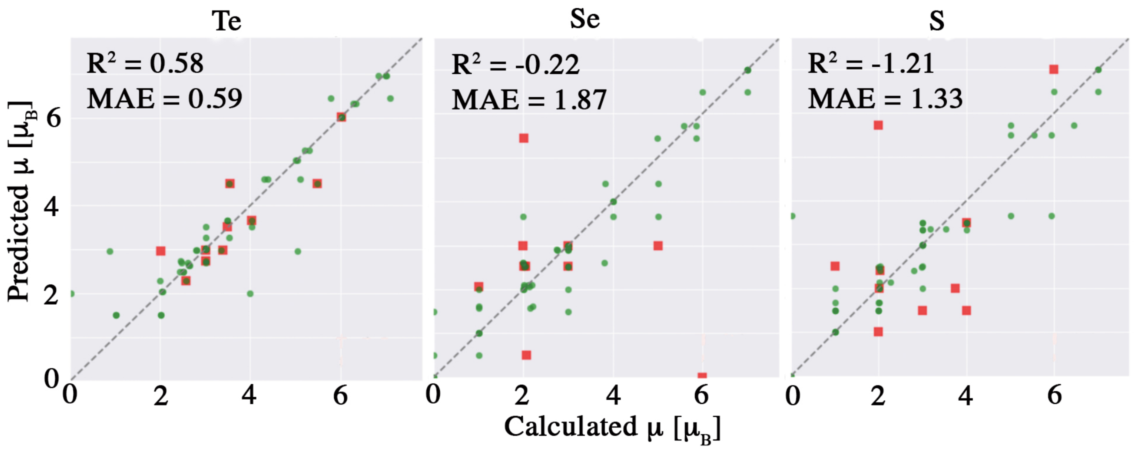

Figure 3. Magnetic moment prediction accuracy for X=Te, Se and S are shown. A data set size, *N* of 66 was used for each panel, for a total of 198 structures. Red squares show test set predictions while green circles show training set predictions.

where 'var.' is the variance and 'max. difference' is the maximum in the set of differences of adjacent pairs. The top twelve descriptors for the formation energy prediction are shown in Table 2. Notice that the descriptor importances for the magnetic moment prediction and the formation energy prediction are different. This is reasonable since the ML models are optimized for a specific target property. The top descriptors for the magnetic moment prediction involve the number of valence electrons and the number of spin up electrons. The importance of these descriptors agrees with physical intuition for the magnetic moment. For instance, we expect that the maximum value for the magnetic moment per unit cell to be the sum of local magnetic moments (i.e. number of spin up electrons) at each site. In the case of the formation energy, the ionization energy and the dipole polarizability are among the top descriptors. This agrees with physical intuition for the formation energy (or chemical stability). For example, those pairs of atoms that have large differences in the ionization energy should easily form strong ionic bonds and thus would lead to a structure of lower (i.e. more negative) formation energy.

5 Magnetic moment predictions for X = Te, Se, S

Magnetic moment predictions for X = Te, Se and S are shown in Fig. S3. ML prediction accuracy decreases as X moves up the group VI column of the Periodic Table from Te to S. The descriptors used in our model do not generalize well across X sites. This may be due to the limited size of our data set or the larger number of degenerate parallel and antiparallel spin configurations for X=Se and S (see Fig. 2(a) in the main text). This may also be due to the larger variation in the magnetic moment per unit cell across B sites for X=Se and S when compared to X=Te structures. In addition, when all composites for X=Te, Se and S are combined, despite the larger training set size, the prediction accuracy is poor. The role of the X site on the magnetic properties is highlighted in Fig. S2. We also find that a dimensionality reduction method similar to PCA, t-stochastic nearest neighbor embedding (t-SNE)[11] demonstrates the importance of the X-sites. t-SNE is used to plot high dimensional (53-dimensional) data describing distances between adjacent A, B and X sites on a two-dimensional manifold (lattice parameters are also included alongside statistics such as the average or variance of the AB, AX and BX distances). The dimensionality reduction results in clustering of points, where three distinct clusters form for X=Te, Se and S. This underscores the impact of the X site and shows the importance of subgroups - using machine learning models to interpolate between

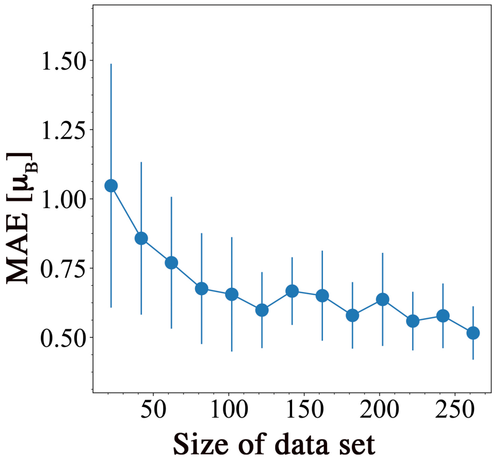

Figure 4. Test set prediction accuracy of the magnetic moment, in terms of mean absolute error (MAE), versus data set size. A training/test set split of 80 to 20 was used.

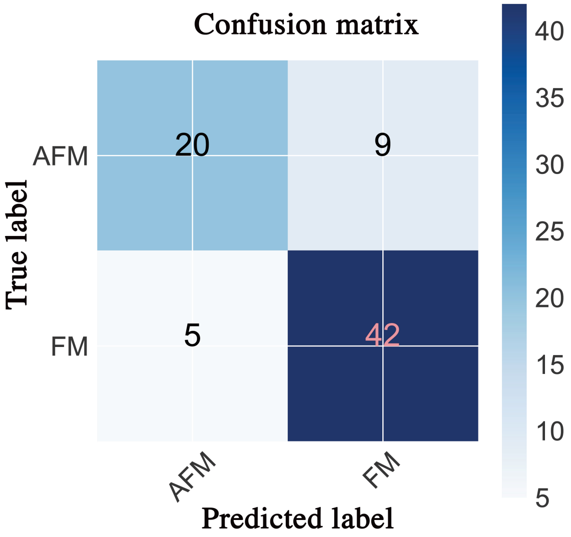

Figure 5. Confusion matrix for magnetic order classification using support vector machines using test set size N=76.

structures within similar regions of chemical space in order to improve prediction accuracy [12]. More sophisticated descriptors will be used in future studies to create more robust machine learning models [13;14;15;16] and improve prediction accuracy for both X=Se and S structures.

6 Prediction accuracy and data set size

In the main text we showed that increasing the size of the training set can increase the prediction accuracy of the formation energy. We extended the investigation of the training set size and prediction accuracy to include the magnetic moment. Initial models were built with data set size, N = 66. We increased the data set size from 66 to 262, fixing X = Te and allowing additional transition metal substitutions at A sites and atomic substitutions at B sites. The prediction accuracy of the test set was calculated for variations in the data set size. The training/test data split chosen here was 80/20. Fig. S4 shows how the test set prediction accuracy varied with the size of the data set.

7 Analysis of magnetic excitation energy

7.1 Classification of magnetic order

We trained a support vector classification model [4] to predict whether an ABX_3 structure was ferromagnetic or antiferromagnetic. The model training and testing was done on 378 structures using a training/test split of 80 to 20 (180 structures were added to

the original data set of 198 structures). We found that the prediction performance improved with increasing data set size. The confusion matrix displayed in Fig. S5 shows the results of the ML classification performed on the test set data. The model predicted 51 structures had FM order, only 42 of which were FM, yielding an accuracy of 82%. The model also predicted 25 structures had AFM order, 20 of which were correct, giving a prediction accuracy of 80%.

7.2 Estimating the Curie temperature

The methods for estimating the Curie temperature T_c for three-dimensional crystal structures is outlined in Ref. 17. The analytical expression for the Curie temperature of three-dimensional crystals is shown below.

$$k_B T_c = \frac{2}{ln(2+\sqrt{3})} J \tag{2}$$

Although this approach has also been used for two-dimensional magnetic materials in the past[18], it does not include the magnetic anisotropy which is a key component of magnetic ordering in two-dimensions owing to the Mermin-Wagner theorem[19]. Ref. 20 describes the importance of the magnetocrystalline anisotropy (MCA) and derives an analytical expression for the Curie temperature, T_c of magnetic two-dimensional materials that incorporates the MCA (see Eqn. 3, 4, 5).

$$T_c = T_c^{Ising} f(\frac{\Delta}{J(2S-1)}) \tag{3}$$

$$\Delta = A(2S-1) + BSN_{nn} \tag{4}$$

$$f(x) = tanh^{1/4}[\frac{6}{N_{nn}} log(1+\gamma x)] \tag{5}$$

where T_c^{Ising} is the critical temperatures for the corresponding Ising model, A is the single ion anisotropy and B is the nearest neighbor anisotropic exchange, N_{nn} is the number of nearest neighbors and $\gamma = 0.033$. See Ref. 20 for details.

The estimates for the magnetocrystalline anisotropy are made by calculating the magnetic anisotropy energy (E_{MA}). E_{MA} is the difference between the DFT total energy of the magnetic configuration with the spins perpendicular to the plane and parallel to the plane. An in-depth analysis of E_{MA} is presented in Ref. 21.

8 Cleavage energy

It is important to determine whether the structures predicted from our analysis are layered materials which are cleavable. In order to estimate this, we determine the cleavage energy of a random sample of ABX_3 structures and compare these values to that of $CrGeTe_3$, a known van der Waals material that has been exfoliated. We find that all the structures we analyzed had cleavage energy comparable to that of $CrGeTe_3$. Consequently, we anticipate that all the ABX_3 structures considered in this work are cleavable layered materials. The results are shown in Table 3. We used the van der Waals DFT-D3 method with spin-orbit coupling included.

Table 3. Cleavage energies $E_{binding}$ (in eV per formula unit) for several ABX_3 structures.

Formula	$E_{binding}$
$Cr_2Ge_2Te_6$	−0.45
Cr_2SiPTe_6	−0.46
$CrNbSi_2Te_6$	−0.46
$CrTiSiPTe_6$	−0.45
$CrYGe_2S_6$	−0.35

Table 4. Dynamic stability results for select candidate structures. Bold-face compound names indicate structures with dynamic stability. Italicized compound names indicate negative phonon frequencies evidencing dynamic instability.

Compound	ENCUT [eV]	Supercell Size	K-Point Grid
**$Cr_2Ge_2Te_6$**	500	3x3x1	3x3x1
**$Cr_2Ge_2Se_6$**	450	2x2x1	10x10x1
**$CrMnSi_2Te_6$**	400	3x3x1	4x4x1
**$CrMoSi_2Te_6$**	450	2x2x1	10x10x1
**$CrSi_2WTe_6$**	450	2x2x1	5x5x1
$CrFeSi_2PS_6$	500	2x2x1	10x10x1

9 Chemical stability

A low formation energy is a necessary but not sufficient condition for chemical stability. Our high-throughput DFT approach uses formation energy as a computationally cheap initial indicator of chemical stability. Further analysis on stability is performed for a subset of promising candidates. This includes determining dynamic stability as well as exploring competing phases.

9.1 Dynamic stability

The formation energy only probes the thermodynamic stability of a pristine structure at absolute zero in the absence of phonons. Computation of the phonon spectrum and looking for positive frequencies evidences that the phases in question would be dynamically stable at low temperatures, whereas negative frequencies in the Brillouin zone indicate dynamic instability, or in some cases, the possibility of charge-density wave formation.

We checked the dynamic stability for a subset of the promising candidates detailed in Supplementary Table 4 using the phonopy Python package[22]. We showed that our baseline compound $Cr_2Ge_2Te_6$ was dynamically stable and present the phonon spectra below. After examining the list of select candidates, we found that $Cr_2Ge_2Se_6$, $CrMoSi_2Te_6$, $CrSi_2WTe_6$, and $CrMnSi_2Te_6$ were dynamically stable. We also tested the candidate $CrFeSiPS_6$ and and found evidence of dynamical instability due to large negative phonons spanning the Brillouin zone.

Some two-dimensional materials exhibit a z-direction acoustic phonon mode which is quadratic in character near the Γ point, which can be notoriously difficult to converge[23;24]; This is because fitting the dynamical matrix to small perturbations of the structure cell requires high precision, as the response due to small perturbations is vanishingly small and numerical noise can result in spurious negative values that do not reflect true dynamical instability.

Consequently, some structures featured an extremely small negative frequency near the Γ point of the Brillouin zone, which we believe to be a numerical artifact as documented in the SI of Ref. 23. $Cr_2Ge_2Te_6$ exhibited a similar minute instability. For that compound, we perturbed the supercell along the direction of the negative frequency and found that the perturbed structure had a higher, not lower, energy and relaxed back to its initial configuration, providing evidence this was a numerical artifact.

Our frozen phonon calculations used displacements of 0.05 Angstrom in radius. We found that in most cases 2x2x1 supercells were sufficient. The k-point grid size used for each calculation is noted in Supplementary Table 4 and were all centered at the Γ point. The path through the Brillouin zone used for all figures corresponds to the path from $(0,0,0) \rightarrow (\frac{1}{2},0,0) \rightarrow (\frac{1}{3},\frac{1}{3},0) \rightarrow (0,0,0)$ or $\Gamma \rightarrow M \rightarrow K \rightarrow \Gamma$[25].

It is possible that going to higher supercell sizes or increasing precision (i.e., via increased k-point sampling, tighter electronic convergence criteria or increased cutoff energy) could result in increased stability, possibly by identifying charge-density wave phases, and/or the elimination of spurious negative modes. A more rigorous test of dynamic stability in vacuum could involve an *ab initio* molecular dynamics simulation at finite temperature, but is beyond the scope of this work. We also note that under experimental conditions, the presence of a stabilizing substrate may support dynamical stability in a given structure[26].

We present the phonon spectra results from the dynamic stability calculations for the subset of candidate structures in Supplementary Fig. S6 through Fig. S11.

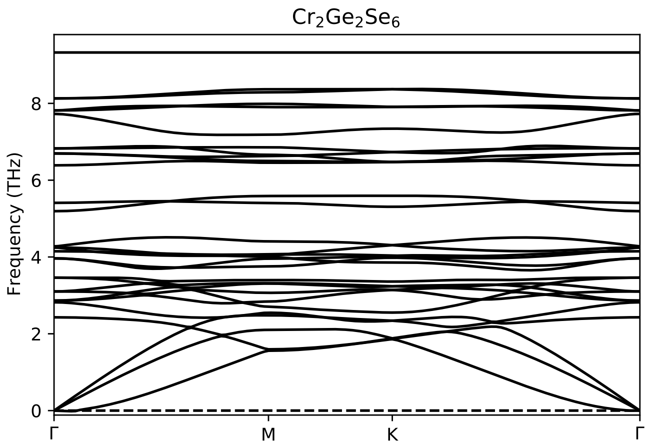

Figure 6

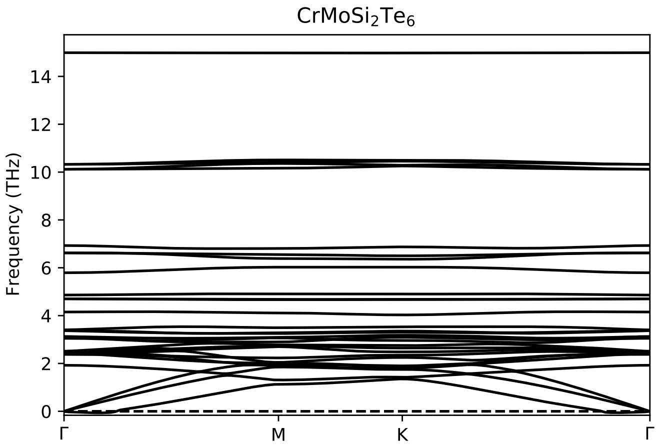

Figure 7

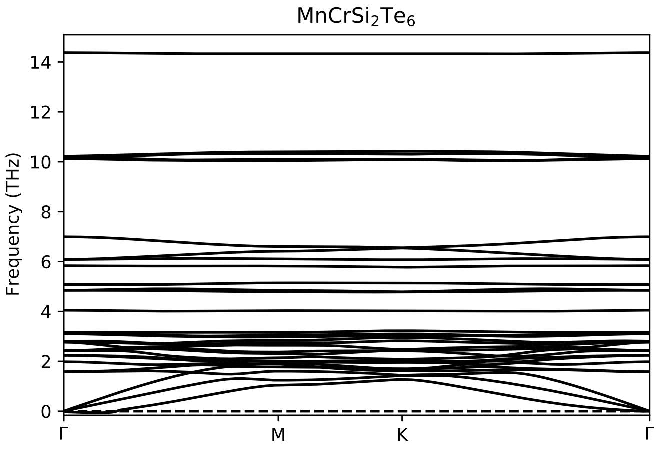

Figure 8

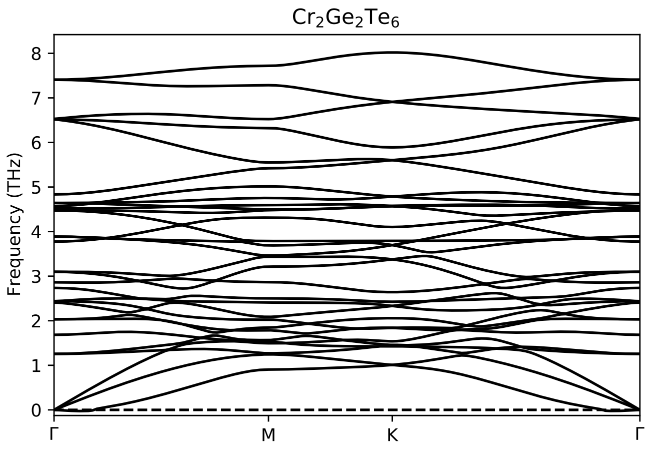

Figure 9

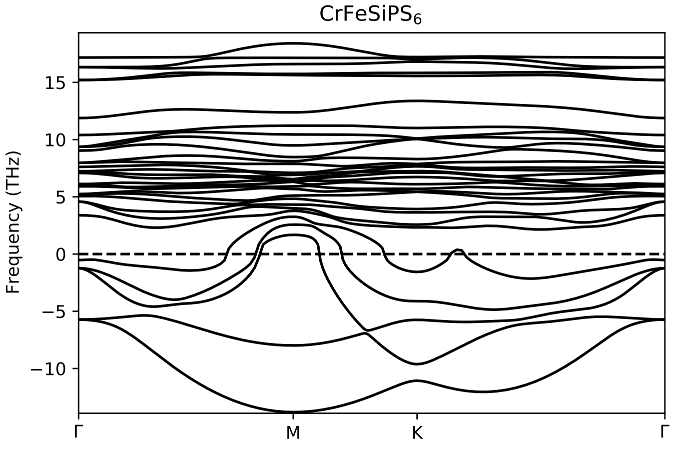

Figure 10

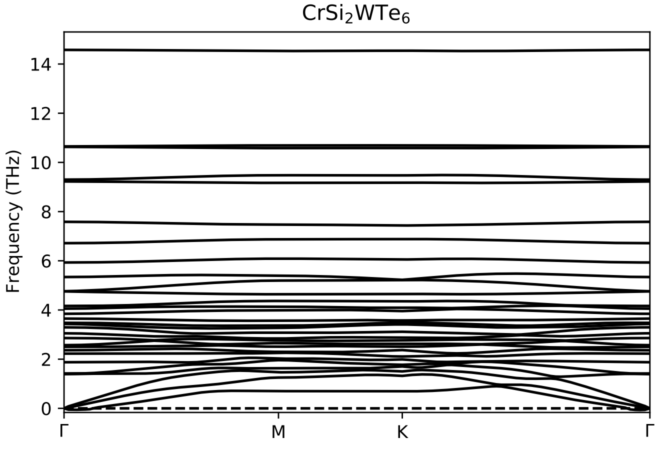

Figure 11

9.2 Competing phases

The goal of materials prediction is to identify materials with desirable properties that are likely to be synthesizable. This includes metastable phases [27], such as the 1T and similar distorted phases of MoS_2, which are higher in energy by 180 to 280 meV/atom compared to the ground state 2H-MoS_2 [28;29;30]. As a test case, we considered a known competing phase of the crystal structure based on $Cr_2Ge_2Te_6$ where the A site substitutions form alternating 'chains' [31]. We performed calculations for ten different example materials ($CrAgP_2S_6$, $CrAgP_2Se_6$, $CrCuP_2S_6$, $CrCuP_2Se_6$, $CrMoSi_2Te_6$, $CrWSi_2Te_6$, $CrMoSiPTe_6$, $CrMnSi_2Te_6$, $CrMnGe_2Se_6$, $CrFeGePSe_6$) assuming they took on the 'chain' structure. For 7 of the materials the energy difference between the structures was less than 15 meV/atom. The largest energy difference found was 68 meV/atom, still significantly lower than the energy difference between 2H-MoS_2 and its related 1T structures. This demonstrates that the materials identified as interesting in this study are likely to be at least metastable and thus worth further detailed theoretical and experimental investigation. Interestingly, the lower energy phase of $A_2B_2X_6$ might also be dependent on the magnetic configuration. This suggests the possibility of an interplay between crystalline phase and magnetic order. Nevertheless, further exploration of all possible competing phases for the entire database of composite structures, including kinetic barriers and synthesis conditions, is beyond the scope of this manuscript.

References

1. Carteaux, V., Moussa, F. & Spiesser, M. 2D Ising-Like Ferromagnetic Behaviour for the Lamellar $Cr_2Si_2Te_6$ Compound: A Neutron Scattering Investigation. *Europhysics Letters (EPL)* **29**, 251–256 (1995).

2. Wildes, A. R., Roessli, B., Lebech, B. & Godfrey, K. W. Spin waves and the critical behaviour of the magnetization in $MnPS_3$. *Journal of Physics: Condensed Matter* **10**, 6417–6428 (1998).

3. Sivadas, N., Daniels, M. W., Swendsen, R. H., Okamoto, S. & Xiao, D. Magnetic ground state of semiconducting transition-metal trichalcogenide monolayers. *Phys. Rev. B* **91**, 235425 (2015).

4. Hastie, T., Tibshirani, R. & Friedman, J. *The Elements of Statistical Learning* (Springer New York Inc., 2001).

5. Ghiringhelli, L. M., Vybiral, J., Levchenko, S. V., Draxl, C. & Scheffler, M. Big data of materials science: Critical role of the descriptor. *Phys. Rev. Lett.* **114**, 105503 (2015).

6. Landrum, G. A. & Genin, H. Application of machine-learning methods to solid-state chemistry: ferromagnetism in transition metal alloys. *J. Solid State Chem.* **176**, 587–593 (2003).

7. Pearson, R. *Chemical Hardness: Applications from Molecules to Solids* (Wiley, 1997).

8. Hansen, K. *et al.* Machine learning predictions of molecular properties: Accurate many-body potentials and nonlocality in chemical space. *The Journal of Physical Chemistry Letters* **6**, 2326–2331 (2015).

9. Cardias, R. *et al.* The bethe-slater curve revisited; new insights from electronic structure theory. *Scientific Reports* **7**, 4058 (2017).

10. Rupp, M., Tkatchenko, A., Müller, K.-R. & von Lilienfeld, O. A. Fast and Accurate Modeling of Molecular Atomization Energies with Machine Learning. *Phys. Rev. Lett.* **108**, 58301 (2012).

11. Hinton, G. & van der Maaten, L. Visualizing Data using t-SNE. *Journal of Machine Learning Research* **9**, 2579–2605 (2008).

12. Goldsmith, B. R., Boley, M., Vreeken, J., Scheffler, M. & Ghiringhelli, L. M. Uncovering structure-property relationships of materials by subgroup discovery. *New J. Phys.* **19**, 013031 (2017).

13. Isayev, O. *et al.* Universal fragment descriptors for predicting properties of inorganic crystals. *Nature Communications* **8**, 15679 (2017).

14. Möller, J. J., Körner, W., Krugel, G., Urban, D. F. & Elsässer, C. Compositional optimization of hard-magnetic phases with machine-learning models. *arXiv:1803.03073* (2018).

15. Gilmer, J., Schoenholz, S. S., Riley, P. F., Vinyals, O. & Dahl, G. E. Neural message passing for quantum chemistry. *arXiv:1704.01212* (2017).

16. Huo, H. & Rupp, M. Unified representation for machine learning of molecules and crystals. *arXiv:1704.06439* (2017).

17. Strečka, J. & Jačšcur, M. A brief account of the ising and ising-like models: mean-field, effective-field and exact results. *Acta Physica Slovaca* **65**, 235 (2015).

18. Zhu, Y., Kong, X., Rhone, T. D. & Guo, H. Systematic search for two-dimensional ferromagnetic materials. *Phys. Rev. Materials* **2**, 081001 (2018).

19. Mermin, N. D. & Wagner, H. Absence of ferromagnetism or antiferromagnetism in one-or two-dimensional isotropic heisenberg models. *Phys. Rev. Lett.* **17**, 1133 (1966).

20. Torelli, D. & Olsen, T. Calculating critical temperatures for ferromagnetic order in two-dimensional materials. *2D Materials* **6**, 015028 (2018).

21. Xie, Y., Tritsaris, G. A., Granas, O. & Rhone, T. D. Data-driven studies of the magnetic anisotropy of two-dimensional magnetic materials. *APS March Meeting*, BAPS.2020.MAR.M39.5 (2020).

22. Togo, A. & Tanaka, I. First principles phonon calculations in materials science. *Scr. Mater.* **108**, 1–5 (2015).

23. Torrisi, S. B., Singh, A. K., Montoya, J. H., Biswas, T. & Persson, K. A. Two-dimensional forms of robust CO_2 reduction photocatalysts. *npj 2D Materials and Applications* **4**, 1–10 (2020).

24. Mounet, N. *et al.* Two-dimensional materials from high-throughput computational exfoliation of experimentally known compounds. *Nature Nanotechnology* **13**, 246–252 (2018).

25. Setyawan, W. & Curtarolo, S. High-throughput electronic band structure calculations: Challenges and tools. *Comput. Mater. Sci.* **49**, 299–312 (2010).

26. Gao, J., Zhang, G. & Zhang, Y.-W. The Critical Role of Substrate in Stabilizing Phosphorene Nanoflake: A Theoretical Exploration. *Journal of the American Chemical Society* **138**, 4763–4771 (2016).

27. Sun, W. *et al.* The thermodynamic scale of inorganic crystalline metastability. *Science Advances* **2**, 1600225–1600225 (2016).

28. Zhao, W. & Ding, F. Energetics and kinetics of phase transition between a 2H and a 1T MoS_2 monolayer – a theoretical study. *Nanoscale* **9**, 2301–2309 (2017).

29. Duerloo, K.-A. N., Li, Y. & Reed, E. J. Structural phase transitions in two-dimensional Mo- and W-dichalcogenide monolayers. *Nature Communications* **5**, 4214 (2014).

30. Kan, M. *et al.* Structures and Phase Transition of a MoS_2 Monolayer. *The Journal of Physical Chemistry C* **118**, 1515–1522 (2014).

31. Susner, M. A., Chyasnavichyus, M., McGuire, M. A., Ganesh, P. & Maksymovych, P. Metal thio- and selenophosphates as multifunctional van der waals layered materials. *Advanced Materials* **29**, 1602852 (2017).
